# Replacement of Monensin with a Proprietary Tannin-Blend Additive in Calf-Fed Holstein Steer Diets

**DOI:** 10.3390/vetsci12020166

**Published:** 2025-02-13

**Authors:** Sydney M. Bowman-Schnug, Luke K. Fuerniss, Joe D. Cameron, Jonathon L. Beckett, Muhammad Ahsin, Stephan van Vliet, Guy D. Hufstedler, Bradley J. Johnson

**Affiliations:** 1 Department of Animal and Food Sciences, Texas Tech University, Lubbock, TX 79409, USA; s.bowman@colostate.edu (S.M.B.-S.);; 2 Mesquite Cattle Feeders, Brawley, CA 92227, USA; joedan@mesquitecattle.com; 3 Beckett Consulting Services, Fort Collins, CO 80524, USA; jbeckett@beefconsulting.com; 4 Department of Nutrition, Dietetics and Food Sciences, Utah State University, Logan, UT 84322, USA; muhammad.ahsin@usu.edu (M.A.); stephan.vanvliet@usu.edu (S.v.V.); 5 Silvateam USA, New York, NY 10022, USA; dhufstedler@silvateam.com

**Keywords:** tannin, monensin, replacement, natural program, Holstein, feedlot

## Abstract

A proprietary tannin blend was evaluated as a potential ionophore replacement considering traits of feedlot cattle growth performance, carcass traits, and health. Holstein steers (*n* = 1450; initial shrunk body weight (SBW) = 141.6 ± 34.2 kg.) were blocked by origin and randomly assigned to receive a finishing diet containing one of two treatments: 1) 14,500 mg per head daily of Silvafeed ByProX (SBPX; ByPro/BX blend, Silvateam S.p.A, San Michele Mondovì, Italy) or 2) 180 mg per head daily of monensin (MON; Rumensin™ 90, Elanco, Greenfield, IN). Steers were on trial for 341 ± 11 days. Pen was the experimental unit (~72 steers/pen), with ten pens per treatment. Data were analyzed in R 4.2.2 and are reported with deads and removals included. Initial SBW tended to be greater (*p* = 0.06) for SBPX steers and was therefore utilized as a covariate for other weighted measures. Treatments experienced similar ADG even though SBPX-supplemented cattle consumed more DM daily (*p* < 0.01); MON cattle had more efficient gain (*p* < 0.01). No difference (*p* > 0.10) was observed for total morbidity and mortality. However, SBPX cattle were more frequently diagnosed with lameness or other illness, while MON cattle experienced greater challenge with respiratory illness and tended to require third pulls more frequently (*p ≤* 0.10). Greater (*p* < 0.05) concentrations of eighteen phytochemical metabolites were recorded in muscle samples from SBPX steers compared to those from MON steers, indicating greater phenolic exposure from tannin-rich supplementation. No difference was observed for carcass traits or liver abscess distributions (*p* > 0.10). Though performance outcomes were not replicated, Silvafeed ByProX could be an option to maintain cattle health in the absence of monensin.

## Simple Summary

With the potential for ionophores to be excluded from diets of naturally fed cattle, tannin-based products could serve as replacements. The objective of this study was to test Silvafeed ByProX, a proprietary tannin-blend product, compared to monensin in terms of growth performance, carcass traits, and health traits in feedlot finishing diets of natural Holstein steers. Health diagnoses differed by treatment, where respiratory disease was less frequent and lameness was more frequent in ByProX-supplemented cattle. The frequency of digestive disturbances was not influenced by treatment. Feedlot performance and gain efficiency were improved with dietary monensin inclusion. Carcass performance was similar regardless of treatment. Plant secondary-derived metabolites were increased in muscle tissues with ByProX treatment. Silvafeed ByProX might maintain cattle health in the absence of monensin.

## 1. Introduction

The discussion of antimicrobial resistance has begun to impact consumer preference and influence the implementation of certain growth-promoting technologies in the cattle feeding industry. Several countries, including the European Union, have already banned livestock growth promotants, placing international attention on their use. One such technology currently under scrutiny is monensin, an ionophore antibiotic that is widely utilized because it moderates dry matter intake (DMI) without depressing weight gain [1,2]. Monensin modifies ruminal microbial communities, which likewise modulates ruminal volatile fatty acid (VFA) production, improves nitrogen metabolism, and leads to feed efficiency improvements [3,4,5,6]. While these performance outcomes are important factors of economic relevance, monensin is also critical to animal health because it effectively controls coccidiosis and reduces the risk of digestive disturbances. These health benefits are especially crucial in calf-fed Holstein steers because of greater time on feed and greater risk of metabolic disorders [2]. With multiple countries and specialty beef programs already requiring monensin exclusion, evaluating potential alternatives is crucial to maintaining both performance and animal health.

Tannins are naturally derived polyphenolic compounds found in many plant varieties commonly consumed by ruminants. While historically viewed as toxic antinutritional factors, tannins exhibit many biological actions that are of interest to food animal production [7]. Plant tannins have a well-recognized affinity for proteins, though their unique structure allows them to bind to and form stable complexes with other dietary polymers as well [8,9]. By precipitating proteins and other nutrients, tannins can decrease ruminal degradation, subsequently improving availability for digestion and absorption within the small intestine [7,10]. Resulting impacts on animal performance have been variable. While some studies have reported an ADG or efficiency improvements associated with tannin supplementation in high-grain [11,12,13] and forage-based [14] diets, other evaluations have reported no effect of tannin inclusion on cattle growth performance [15,16,17]. Health is also a crucial consideration for conventional additive replacement, and tannins have been known to possess other properties demonstrating antimicrobial, antiviral, antiparasitic, and antioxidant-like effects [7,10]. The extent of tannin-mediated performance and health outcomes is considered highly variable based on tannin type and origin [18].

Monensin and tannins are both recognized for their ability to alter rumen fermentation and metabolism of cattle. While the mechanisms are unique, there is potential that these similarities may allow a proprietary tannin-blend product to mimic performance outcomes generally attributed to ionophores. Moreover, specific interest has been expressed in the potential ability of this product to improve cattle health and limit the necessity of antibiotic treatment. Therefore, the objective of this experiment was to test Silvafeed ByProX, a proprietary tannin-blend additive, compared to monensin for growth performance, carcass, and health traits in feedlot finishing diets of calf-fed Holstein steers.

## 2. Materials and Methods

### 2.1. Animal Care and Use

This experiment was a collaborative effort conducted at a commercial feedlot near Brawley, CA. All research followed the guidelines stated in the Guide for the Care and Use of Agricultural Animals in Agricultural Research and Teaching [19].

### 2.2. Animals, Diet, and Experimental Design

A total of 1450 Holstein steers with an average initial shrunk body weight (SBW) of 141.6 ± 34.3 kg (Mean ± Range) were blocked by origin (10 blocks; 145 steers per block) and randomly assigned to receive one of two treatments (~72 steers per treatment block; *n* = 10 pens per treatment). Upon feedyard arrival, steers were given free-choice access to hay and rested for a minimum of 48 h before initial processing. At processing, steers received a pour-on for control of internal and external parasites and were vaccinated for respiratory (Pyramid 3 LPH, Boehringer Ingelheim, Duluth, GA, USA; Nasalgen, Merck, Madison, NJ, USA) and clostridial (Vision 7 with SPUR, Merck, Madison, NJ, USA) diseases. Additionally, steers received a macrolide antibiotic (Increxxa, Elanco, Greenfield, IN, USA) for the control of bovine respiratory disease (BRD) and a probiotic drench (Lactipro NXT, MS Biotec, Wamego, KS, USA).

The feedyard allowed for approximately 16.1 square meters of pen space and 5.6 square meters of covered shade space per steer. Each pen also included 30.5 m of concrete bunk space and a concrete water trough. Pens were allocated such that the same treatment shared a feed bunk line, which was done to minimize the risk of incorrectly administering treatment diets. Both treatments shared a drovers alley and pens within block were assigned such that they were immediately adjacent to one another across the drovers alley. Steers received the same diet throughout the course of their 357-day feeding period. An adaptation period was not utilized because cattle were sourced directly from a calf ranch where they were receiving a high energy grower ration with very little roughage. While the feedlot finishing diet was more energy dense, it also contained a greater amount of roughage, which was believed to negate the need for transition diets. The feedlot finishing diet formulation is shown in Table 1. It was composed of 60% steam-flaked corn and 13% roughage and reached a Net Energy for gain (NE_G_) of 1.47 Mcal/kg. Micro ingredients supplied on a per-head basis included Availa Zn 120 (3000 mg/hd/d; ZinPro, Eden Prairie, Minnesota), Bovamine Defend Plus 20 k (50 mg/hd/d; Chr. Hansen, Milwaukee, Wisconsin), and vitamins A (5225.12 IU/kg) and D (522.52 IU/kg). Cattle were randomly assigned to receive finishing diet containing either: (1) 14,500 mg (~0.15% of diet DM) per head daily of an experimental proprietary combination blend of condensed tannin extract from quebracho (*Schinopsis lorentzii*), hydrolysable tannin extract from chestnut (*Castanea sativa*) and carriers from cereals rich in saponins, Silvafeed ByProX (SBPX; ByPro/BX blend, Silvateam S.p.A, San Michele Mondovì, Italy) or (2) 320 mg per head daily of monensin (MON; Rumensin™ 90, Elanco, Greenfield, IN, USA). Steers were fed an average of 341 days on the trial diet before being transported 378.2 km for harvest at a commercial beef processing plant in Tolleson, Arizona.

### 2.3. Feedlot Performance Measures

Steers were weighed the morning before trial initiation and a 4% shrink was applied to determine initial SBW. There was a tendency for SBPX cattle to weigh more, so initial SBW was used as a covariate for all other weighted measures. Daily feed deliveries were recorded and used to determine daily dry matter intake (DMI). Additionally, the average variation (CV, %) in feed deliveries was analyzed separately. Steers were again weighed prior to shipping for harvest and a 4% shrink was applied to calculate final SBW. Final SBW and DMI were utilized to calculate average daily gain (ADG) and efficiency (G:F).

### 2.4. Measures of Health Response

Health data were collected throughout the feeding period. An average gap period of 16 ± 3 days existed between cattle arrival and trial start. All mortalities and removals made before dietary treatments were applied have been excluded from the study. Additionally, all treatments made prior to trial start were also excluded. Initial pulls were defined as all cattle removed from their home pen and transported to a hospital because of suspected disease or defect. Repulls were cattle that were pulled again for any further treatment. Neither initial pulls nor repulls includes steers that were removed as suspected bullers. Bullers were identified as animals that underwent excessive riding or imitated bull-like behavior, causing a reduction in performance. All health-related pull decisions and resulting diagnoses were made by trained individuals at the feedyard and treatment plans were executed according to yard protocol. These individuals were blinded to the study. Costs associated with treatment, including chute charges and medicine costs, were recorded and analyzed. Railers were identified as any steers that appeared unthrifty and were deemed unfit to remain in the feedyard. All mortalities were recorded and necropsies were performed by a trained individual to determine cause of death. Cattle that were removed from their home pen due to health treatment that nullified their eligibility for the natural program (natural program fallouts) or cattle that were railed were likewise not considered in the study following the date of removal. The remaining steers, which were shipped for harvest in the intended natural program, are considered natural program candidates and were likewise eligible for premiums associated with the natural program.

### 2.5. Carcass Performance Measures

Steers were shipped to a commercial beef processing plant in Tolleson, AZ, for harvest based on a visual assessment of finish. Both treatments within each block were shipped on the same date. Lot tags were noted to ensure that all cattle and resulting carcasses were properly identified within respective treatment groups. Following removal of the gut mass, livers were scored according to Elanco’s liver abscess classification system by trained individuals from Texas Tech University. Hot carcass weight (HCW) was recorded after final trim. 12th rib fat thickness was measured after carcasses underwent a 24 h chill. At this time, imprints of the Longissimus dorsi muscle were also taken and later traced and measured with an approved grid to determine ribeye area (REA) in centimeters squared. USDA quality (QG) and yield grades (YG) were evaluated by a certified USDA grader.

### 2.6. Phytochemical Quantification

A subset of USDA Choice carcasses (*n* = 18 per treatment) were randomly selected and muscle samples estimated to weigh between 10 and 15 g were taken from the neck. These samples were stored in individually labeled collection bags and frozen using dry ice. Following collection, samples were shipped to Utah State University for phytochemical quantification. Muscle samples were pulverized under liquid nitrogen, and approximately 200 ± 5 mg of each sample was extracted with 1.2 mL of a solution containing internal standards (4-hydroxybenzoic acid-^13^C_6_, apigenin-d_5_, benzoic acid-d_5_, genistein-d_4_, phenol-^13^C_6_, quercetin-D_3_, resveratrol-^13^C_6_, and trans-cinnamic acid-d_7_) at concentrations of 1, 1, 5, 1, 5, 1, 1, and 5 µM, respectively in a MTBE:MeOH (2:1, *v*:*v*) solution. The samples were homogenized for 5 min using a tissue lyser set at 30 oscillations/sec (Qiagen Retsch Tissue Lyser II, Germantown, MD, USA). Proteins were precipitated at −20 °C for 2 h, and the supernatant was collected after centrifuging the samples at 18,000 rcf for 10 min at 4 °C (Eppendorf 5145 R, Hamburg, Germany). The MTBE was then removed by phase separation through the addition of 0.8 mL of water, and the aqueous methanolic extract was dried under a nitrogen stream (Biotage, TurboVap^®^ LV, Uppsala, Sweden). The dried extracts were reconstituted in 100 µL of a 50% methanolic solution containing 0.1% formic acid.

A 5 µL sample was separated using a Kinetex Core-Shell F5 100Å (2.1 mm × 150 mm, 2.6 μM) column (Phenomenex, Torrence, CA, USA) using a binary gradient of water (Mobile Phase A) and acetonitrile (Mobile Phase B), both containing 0.1% formic acid. The flow rate was set at 200 µL per min, starting with 5% B, which linearly increased to 95% from 2.1 to 14 min; then B was decreased to 5% within a minute, and the system was equilibrated for 4 min. The column oven temperature was set at 30 °C, while the samples were kept at 4 °C. The analysis was performed with a Ultra High-Performance Liquid Chromatography (UHPLC) system (Nexera 40 Series, Shimadzu, Japan) combined with a Sciex Hybrid Triple Quad 7500 Low Mass Spectrometer (Framingham, MA, USA). The ion source was operated at temperature of 550 °C and spray voltage of 1600 v, Gas-1 at 40 psi, and Gas-2 at 60 psi for both positive and negative scheduled multiple reaction monitoring (MRM) methods, as described previously [20]. The cycle time was set at 1000 msec, with dwell times ranging from 3 to 250 ms. Double blank (100% methanol) and internal standard blank samples were run every 20 samples for quality control purposes. Unlabeled external standard mixes were run in parallel to the samples with known concentrations (ranging from 6250 nM to 0.38147 nM using a 1-fold serial dilution) to allow for quantitation of compounds. Sciex OS 3.1 software (AB Sciex, Framingham, MA, USA) was used to acquire and analyze chromatographic data. Peaks were integrated using area-under-the-curve and normalization was performed using isotopically labeled standards to account for any loss of material during sample preparation. In case of missing values for compounds, minimum values were imputed on a per group basis given that the compound was detected in at least one sample in that group. In case no values were detected for a metabolite (below lowest level of quantitation; LOQ) in either group, zero values were imputed to allow for statistical analysis.

### 2.7. Statistical Analysis

Statistical analyses were performed in R version 4.2.2 (The R Foundation, 2022, Indianapolis, IN, USA), except for the plant-secondary metabolite analysis, which were performed using MetaboAnalyst 6.0. Pen was considered the experimental unit, with ten pens per treatment. Functions from the dplyr package [21] and purrr package [22] were used for data merging and initial calculations. The dplyr [21], forcats [23], and tidyr [24] packages were utilized to summarize data from the individual level to the lot level. One-way ANOVA analysis was conducted for all quantitative traits. Assumptions of linear models (variance equality and homogeneity, error normality, freedom from outliers, etc.) were first tested using functions from the car package [25] and the rstatix package [26]. Linear models were constructed using the lmer function of the lme4 package [27,28]. Functions of the car package [25] developed the ANOVA models. For all weighted measures, initial shrunk body weight was included as a covariate. Animals were separated in 10 blocks by arrival date. Categorical data distributions were tested by ordinal logistic regression. The initial mixed model was constructed using the clmm function of the ordinal package [29] and analysis was conducted using functions of the RVAideMemoire package [30]. Estimated marginal means were calculated using the emmeans package of R [31]. Significance was assessed by ANOVA testing of the main effect of treatment. Phytochemicals were tested using Wilcoxon rank-sum test with false discovery rate (FDR) adjustment. Statistical significance was evaluated compared to an α of 0.050. Tendencies were considered when 0.05 < *p* ≤ 0.10. Data visualizations were built in R using the ggplot2 package [32] and MetaboAnalyst 6.0.

## 3. Results

### 3.1. Performance Responses

Data are reported on a deads-and-removals-in basis. All cattle were on trial an average of 341 ± 2.7 days (*p* = 0.99; Table 2). Cattle supplemented with SBPX had an average 358 days on feed (DOF), while MON cattle had 357 DOF (±2 days; *p* = 0.19). A tendency was observed for SBPX steers to weigh more upon study enrollment (*p* = 0.06; 143 vs. 140 kg). Therefore, initial SBW was used as a covariate for all other weighted measures. Even though SBPX cattle consumed 0.28 kg more DM per day on average (*p* < 0.01), both groups had similar final SBW and ADG (*p* > 0.10). MON cattle had more efficient gain (*p* < 0.01). Likewise, SBPX cattle had greater daily cost by 14 cents compared to MON cattle (*p* < 0.01). Numerically, SBPX cattle had 1.6 percentage points greater natural program candidates and 2.0 percentage points fewer program fallouts (*p* > 0.10).

### 3.2. Health Responses

Total morbidity was similar (*p* = 0.82; Table 3) for both treatment groups. While first and second pull frequencies were similar (*p* > 0.10) across treatment groups, there was a tendency (*p* = 0.10) for a greater third pull frequency for MON lots. Still, there were no detectable differences in the percent of cattle that required first, second, or third treatment protocols (*p* > 0.10). A numeric USD 1.58 per head treated advantage in treatment cost favored the SBPX group but was not significant (*p* = 0.35). There was no difference in buller frequency, railer frequency, or total mortality (*p* > 0.10).

**Figure 1:**
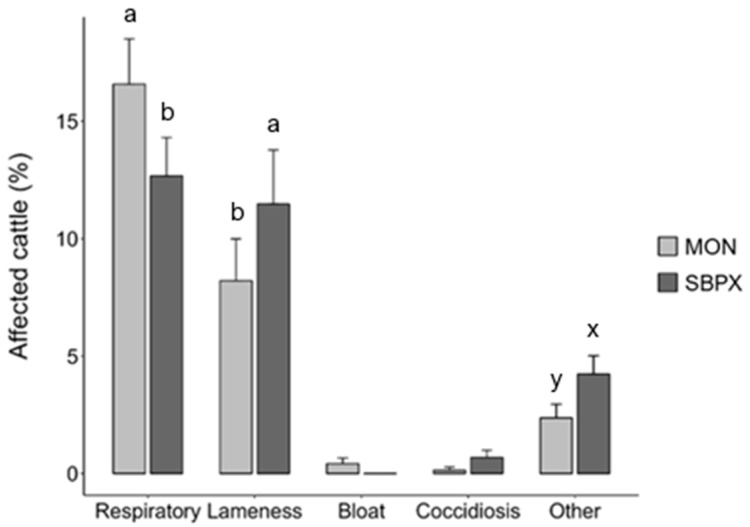
The frequency of illness diagnoses associated with health challenges of Holstein steers supplemented with either a proprietary tannin-blend additive (SBPX; 14,500 mg/hd Silvafeed ByProX) or monensin (MON; 320 g/hd Rumensin™ 90). Letter superscripts are included to illustrate means that are different by pairwise comparison by either *p* ≤ 0.05 (a, b) or *p* ≤ 0.10 (x, y). “Respiratory” refers to an illness that afflicted the upper respiratory tract (*p* = 0.04). “Lameness” refers to afflictions that impacted cattle mobility, such as foot rot and joint illness (*p* = 0.03). “Bloat” refers to ruminal tympany and includes gaseous and frothy varieties. “Coccidiosis” refers to cattle with symptoms consistent with the parasitic disease caused by *Eimeria* spp. of protozoa. “Other” refers to any other treatments made for clinical symptoms of illness not representative of the other diagnostic categories (*p* = 0.06).

While total morbidity did not differ, minor differences in the frequency of diagnosed ailments were detected between treatment groups (Figure 1). Respiratory disorders impacted 3.9 percentage points more (*p* = 0.04) MON cattle; SBPX lots were more frequently (*p* = 0.03) afflicted with lameness and tended (*p* = 0.06) to be pulled more frequently for other reasons. There was no difference (*p* > 0.10) in the occurrence of bloat or coccidiosis between treatments.

### 3.3. Carcass Responses

There was no recorded difference (*p* > 0.10; Table 4) for HCW, 12th rib fat, or REA. Additionally, frequency distributions for QG, YG, and liver abscess prevalence were not affected by dietary treatment (*p* > 0.10).

### 3.4. Phytochemical Status

A full list of metabolites and their concentrations is found in Table 5. A total of 43 phenolics were detected in muscle from SBPX steers and 37 phenolics were detected in muscle from MON steers. The concentrations of 32 phenolics were greater in the SBPX group, with 18 showing a significant (*p* < 0.05) increase. One compound was significantly (all, *p* < 0.05) higher in the MON group. Additionally, the metabolite profiles of the SBPX cattle had lesser variation. Compounds found to be enriched in the SBPX samples compared to monensin included various phenolic-derived metabolites such as 2,6-dihydroxybenzoic acid and salicylic acid, and flavonoid-derived metabolites such as hyperoside and sophoricoside, indicating that supplementation with this proprietary blend of tannins increased plant-derived secondary metabolites in the tissue of SBPX animals as a result.

## 4. Discussion

This work evaluated the effects of a proprietary tannin-blend additive and monensin on feedlot cattle growth performance, carcass traits, and health traits. It is important to note that, as a limitation of the experimental design and considering that monensin feeding is standard practice in U.S. cattle feeding operations, a negative control was not utilized in this study. Outcomes are meant to compare the tannin blend to monensin directly and should be interpreted accordingly. To the best of the authors’ knowledge, this is one of the first studies to compare this proprietary blend of tannins directly to monensin in long-fed Holstein cattle. The only other known study to employ similar constructs has been reported by Carvalho et al. [15]. Other trials have utilized basal diets that may or may not include monensin, but this is generally kept consistent between control and tannin-containing treatments.

### 4.1. Performance Responses

Performance outcomes are for Holstein steers; therefore, outcomes may vary in other cattle types. Both initial shrunk body weight (SBW) and days on feed (DOF) in this work are consistent with a typical calf-fed management system in the Desert Southwest, in which calves transition from the calf ranch to the feedlot early in life. These calves are already accustomed to feed bunks and to consuming high-concentrate diets, so a diet adaptation period was not necessary [2]. Likewise, steers in the present study received the same basal diet throughout the entirety of the feeding period. However, trial induction was delayed, allowing for adjustment to the feedlot setting.

High levels of dietary tannins often result in a DMI decrease, potentially influenced by decreased palatability due to tannins’ astringent flavor [33,34] or a reduced rate of ruminal digestion and passage [7]. However, lower concentrations of tannins have less detrimental impacts on intake. While Koenig et al. [35] observed a tendency for DMI to decrease with inclusion of 3.5% CT extract in beef cattle finishing diets, no impact was reported at either 1.2% or 2.4%. Low levels of dietary tannin supplementation have repeatedly resulted in increased DMI in feedlot cattle. In two consecutive, 84 d finishing trials evaluating tannin level and source, Rivera-Méndez et al. [11] observed a linear tendency for DMI to increase with level of supplementation and for tannin inclusion to increase DMI. The highest inclusion utilized was 0.6% (DM basis)—much lower than that evaluated in [35]. Additionally, a 50:50 blend of quebracho and chestnut tannins resulted in the greatest DMI increase compared to either fed individually [11]. Tabke et al. [17] reported that supplementation of the same tannin blend promoted intake during the first half of the feeding period relative to a negative control. This is the exact combination of tannin-types and source utilized in the present study. Therefore, the 3.8% increase in DMI (*p* < 0.01) associated with ByProX feeding is consistent with previous findings. This outcome, however, should be regarded with reservations, as it is evaluated in comparison to cattle being fed monensin, a known intake-moderator [1,3]. On average, monensin results in a 3.0% depression in DMI [1]. Consequently, the 3.8% increase in SBPX DMI (*p* < 0.01) could be attributed to either a lack of monensin or a true effect of the tannin.

Even though greater DMI was observed among the SBPX cattle, less variation in daily feed deliveries, which reflect intake, was noted for cattle supplemented with the proprietary tannin blend. A similar response was previously noted in the first 28 days of a feedlot finishing period for Nellore bulls, where supplementation of a tannin-saponin blend additive with monensin limited DMI fluctuation compared to monensin fed alone [36]. It was hypothesized that inclusion of tannin-containing additives may more consistently protect against acidotic ruminal conditions, which in turn would limit feed intake variation [37]. In the present study, intake variation differed by treatment throughout the whole feeding period. However, the pen-level metrics recorded in this study do not allow for elucidation of potential individual-level mechanisms. Intake variation deserves more attention in future evaluations of tannin-blend supplementation.

With similar final SBW and ADG, efficiency differences were evident in this trial. Tannin inclusion rates at much higher levels in previous studies have not been associated with efficiency improvements compared to negative controls [33,38]. Koenig et al. [35] observed a tendency for 2.5% CT inclusion (DM basis) to actually depress gain-to-feed ratio (G:F) in high-protein feedlot finishing diets. However, this outcome could be correlated to the high dietary concentration of tannin utilized. At more conservative levels, Krueger et al. [16] noted no difference in G:F for cattle consuming high-grain diets supplemented with 1.5% (DM basis) of tannin extracts, regardless of dietary tannin source. Rivera-Méndez et al. [11] observed increased gain efficiency in an initial trial evaluating CT-tannin dose effects, but this outcome was unable to be replicated in a subsequent evaluation of tannin source at similar doses. Monensin has a long-established and proven reputation for improving efficiency. Historically, monensin has resulted in an average 6.4% improvement in feed efficiency (F:G), with a 2.5 to 3.5% improvement observed in studies conducted within the last three decades [1]. In the present study, cattle supplemented with monensin experienced more efficient gain than cattle supplemented with the proprietary tannin blend.

### 4.2. Health-Related Outcomes

#### 4.2.1. Phytochemical Status

Tannins and their downstream metabolites have long been recognized and utilized in mammalian health for their medicinal properties [39]. The higher concentration of phenolics in the ByProX muscle tissue samples aligns with existing literature, suggesting that phenolic-derived metabolites are absorbed into biological systems [40]. Dietary polyphenols are modified by microbiota and first-pass metabolism, resulting in different forms in the blood and tissues compared to the original dietary source found in plants [41,42]. Consequently, higher levels of hydroxybenzoic acids and hydrolyzable tannins, including 2,6-dihydroxybenzoic acid, 3,4-dihydroxybenzoic acid, salicylic acid, 4-ethylphenol, and ellagic acid were observed in the ByProX group (Table 5). These metabolites are typically considered biomarkers of a diet higher in phenolics [43], and both chestnut [44] and quebracho [45] are considered rich in phenolic acids such as benzoic acid and its metabolites. Additionally, higher amounts of tannic acid metabolites epicatechin gallate and epigallocatechin gallate were detected in the ByproX group, which similarly reflects their presence in the chestnut-derived product fed to cattle in this work [46]. 

#### 4.2.2. Health Responses

The numeric advantage in natural program candidates and lower rate of fallouts associated with SBPX inclusion is attributable to variation in morbidity, as there was no difference in mortality or railer incidence. Rather, MON cattle tended to require a third pull more frequently, which indicates an additional opportunity to require treatment and correlates with the numerically greater treatment cost associated with MON lots. Treatment with antimicrobials excluded cattle from the natural program, designating them as a fallout. At the time cattle were marketed, natural program candidates were receiving an average 7.00 USD/cwt premium above fallout cattle that had to be marketed in the conventional program. However, this does not adequately account for the cost of forfeiting performance advantages associated with conventional technologies, but then also failing to secure premiums to offset these natural consequences. Likewise, health outcomes associated with addition of this proprietary tannin blend hold significant economic relevance.

The explanation for this third pull difference may be further associated with the variation in predominant health diagnoses between treatments. MON cattle were more frequently diagnosed with respiratory illness (*p* = 0.04), and 79% of all third pulls were made for respiratory-related ailments. Tannins have reported inhibitory action on many pathogenic bacteria, such as *Escherichia coli* O157:H7, *Clostridium*, and *Salmonella* [47,48,49]. This inhibitory action has been known to extend to several agents implicated in the bovine respiratory disease (BRD) complex, including *Mannheimia haemolytica*, *Mycoplasma bovis*, and bovine adeno-associated virus [50,51,52]. Therefore, the lesser frequency of diagnosed respiratory ailments within the SBPX cattle (*p* = 0.04) could be associated with antibacterial and/or antiviral-like properties of the proprietary blend of tannins supplemented in this study. However, these properties are highly variable based on tannin type, source, and concentration [48]. Additional research is needed to evaluate the potential inhibitory effect and mode of action for the proprietary tannin-blend product supplemented in the present study on pathogenic agents of interest.

Differences in the frequency of lameness and other diagnoses may be related more to individual animal variation rather than an effect of treatment. Lameness incidence is rarely mentioned in tannin research, and a tannin-containing blend product reported no impact on lameness in finishing cattle [53]. While there is no evidence to support that tannin feeding could cause lameness, monensin has been evaluated as a potential preventive with varying outcomes. While Heuer et al. [54] noted that dairy cows supplemented with monensin prepartum had a lower case and leg incidence of non-infectious lameness, other evaluations found that treatment with monensin did not alter the risk of cows developing lameness [55,56,57]. Inconsistent findings leave the verdict up for discussion—likewise, lameness prevention does not register on monensin’ s list of positive feedlot impacts. Future attention should be paid to feedlot cattle lameness incidence in the presence of tannin or absence of monensin to rule out unintended detriments.

On the other hand, the ability to reduce bloat occurrence is one of the more frequently documented tannin-associated health benefits. In pasture situations, tannin-containing forages are considered “bloat-free” and can be incorporated in richer forage offerings, like alfalfa or clover, to control bloat [7,58,59]. This effect is particularly prominent in condensed tannins, which exhibit preventive actions at concentrations as low as 1–5 g/kg [59]. While the specific mode of action has yet to be delineated, proposed mechanisms include reduction in protein solubility through precipitation during chewing and rumination, destabilization of the proteinaceous foam associated with frothy bloat, and inhibition of slime-producing ruminal bacteria [58,60,61]. Monensin has also been recognized as a bloat-mitigator, with the ability to decrease both the occurrence and the severity of bloat [3,62]. These noted similar impacts make it unsurprising that bloat occurrence did not differ by treatment in the present study (*p* = 0.97).

Originally recognized as a poultry coccidiostat, monensin is still labeled for the prevention and control of coccidiosis caused by *Eimeria bovis* and *Eimeria zuernii* in cattle at all production stages (Rumensin^®^, Elanco, Greenfield, Indiana) [63,64]. Exclusion, therefore, makes coccidiosis incidence a health risk of particular concern, especially in newly received calves. Tannins may be effective in coccidiosis prevention, as they have been long utilized for the natural control of parasites. Condensed tannins, specifically quebracho and sericea lespedeza, have been recognized for their potent ability to reduce fecal egg count, larval development, and worm burden in small ruminants [65,66]. Parisi et al. [67] found that quebracho (CT) and chestnut (HT) tannin extracts were both capable of reducing *Eimeria* spp. oocyst excretion. This potential for tannins to also work as coccidiosis-preventatives is consistent with no differences in occurrence in the present study.

### 4.3. Carcass Responses

Carcass outcomes associated with dietary tannin inclusion are highly variable, with tannin source, cattle type, and diet potentially influencing results. In the present study, carcass characteristics were not affected (*p* ≥ 0.20) by dietary treatment. These findings coincide with previous research [15,17,35] in which carcass measures such as hot carcass weight (HCW), longissimus muscle area (LM area), quality grade, and liver score were unaffected by dietary supplementation of either mimosa (CT) tannin (2.50% DM basis, [35]) or a quebracho (CT)–chestnut (HT) tannin blend (0.15% DM basis, [15]; 0.30% or 0.60% DM basis, [17]). In contrast, Camacho et al. [68] observed a 25.5 kg increase in HCW and a 3.4 cm^2^ increase in LM area (adjusted for final BW difference) for *B. indicus* × *B. taurus* bull calves supplemented with 0.32% (DM) of the same quebracho–chestnut tannin blend (ByPro). When compared directly to monensin addition in feedlot finishing diets of Nellore and Nellore × Angus bulls, ByPro supplementation resulted in an approximate 9 kg increase in HCW [13]. Improvements in HCW were recorded for ByPro versus a negative control and for the combination of ByPro and zilpaterol hydrochloride compared to the beta agonist alone [69]. BX (the proprietary tannin blend plus saponins) inclusion has also been noted to increase the HCW of bulls finished in both feedlot [70] and pasture [14] settings.

The major defining difference between this study and other studies is the utilization of intact bulls, as the aforementioned studies were conducted on implanted steers. Additionally, Latin American systems often target shorter feeding periods and feed diets that maximize lean carcass weight, while cattle feeding in the United States has systemic aims towards improved carcass quality, often associated with increased fat deposition. Diet characteristics likewise differ significantly and might also influence results. Camacho et al. [68] provided a basal diet of dry ground corn, canola meal, and dry distillers grain, and the ration utilized by Nascimento et al. [13] consisted primarily of cracked corn, soybean meal, and sugarcane bagasse. These are very different from the steam-flaked corn-based ration utilized in the present study and by both Carvalho et al. [15] and Tabke et al. [17]. Differences in dietary protein level and source (true protein vs. non-protein nitrogen) along with variation in grain processing method and subsequent carbohydrate availability could play a role in outcome variation.

## 5. Conclusions

Feedlot performance is a concern when monensin is excluded from finishing diets, and ByProX inclusion did not replicate the same efficiency advantages. Economic attention should be paid to greater DMI and daily cost when cattle do not receive monensin. Future evaluations should consider potential applications of this proprietary tannin blend in conventional systems, where it may function synergistically with ionophores and where health and muscle growth, rather than fattening, are the primary focus. Monensin also plays a critical role in mitigating digestive disturbances—a characteristic which is especially crucial when feeding Holsteins. Though ByProX did not moderate DMI, it did stabilize intake variability, and bloat occurrence did not differ between ByProX- and monensin-supplemented cattle, which could make it a valuable tool in situations where monensin cannot be used or where days of use are limited. In natural or organic marketing programs that restrict the use of antibiotics, tannin blends could serve as safe replacements for monensin as one potential means of limiting economically problematic losses from bloat deaths. The lesser frequency of respiratory illness amongst tannin-supplemented lots suggests that ByProX might possess antimicrobial-like properties, which could be facilitated through greater phenolic accumulation in tissue, though further research should be conducted to evaluate this possibility. Health outcomes from the present study suggest that Silvafeed ByProX may be able to maintain cattle health in the absence of conventional additives like monensin and may serve to help limit antibiotic necessity.

## Figures and Tables

**Table 1:** Formulated dry matter ingredient inclusion in basal diet.

	Finishing Diet
Formulated DM inclusion, %	
Steam-flaked corn	59.43
Roughage blend	12.96
Dried distillers grain	8.14
Bakery waste	11.40
Fat blend	4.26
Protein supplement	1.43
Mineral supplement ^1^	2.37
Chemical analysis	
Dry matter, %	82.90
Crude protein, %	13.31
Acid detergent fiber (ADF), %	7.92
Neutral detergent fiber (NDF), %	17.29
Physically effective NDF, %	8.28
Crude fat, %	8.65
NEM, Mcal/kg	2.34
NEG, Mcal/kg	1.47

^1^ Supplement was formulated to include 25.842% calcium, 15.000% salt, and 1.078% magnesium.

**Table 2:** Feedlot performance of Holstein steers supplemented with either a proprietary tannin-blend additive (SBPX; 14,500 mg/hd Silvafeed ByProX) or monensin (MON; 320 mg/hd Rumensin™ 90).

	Treatment			
	MON	SBPX	SEM ^1^	*p*-Value ^2^
Pens, *n*	10	10		
Days on trial	341	341	2.7	0.99
Total days on feed	357	358	2.0	0.19
Initial SBW, kg	140 ^y^	143 ^x^	3.6	0.06
Final SBW, kg	569	570	2.7	0.82
DMI, kg/day	7.34 ^b^	7.62 ^a^	0.060	<0.01
ADG, kg	1.26	1.25	0.006	0.71
Gain:Feed	0.17 ^a^	0.16 ^b^	0.001	<0.01
Daily cost ^3^, USD	3.97 ^b^	4.11 ^a^	0.049	<0.01
Daily per head DM deliveries CV, %	20.95 ^a^	19.75 ^b^	0.858	<0.01
Natural program fallouts ^4^, %	8.12	6.09	1.371	0.13
Shipped for natural program ^5^, %	89.57	91.13	1.619	0.31

^1^ The largest standard error of the mean. ^2^ Tested as the ANOVA significance of the main effect. ^3^ Daily cost references are cost-specific to feed and other daily production inputs but do not include costs associated with processing or health treatment. ^4^ Cattle that required antibiotic health treatment that nullified their eligibility to be harvested in the intended natural program. ^5^ Cattle that remained eligible to be harvested in the intended natural program and that were likewise eligible for associated premiums. ^a, b^ Means without a common superscript are different by pairwise comparison (*p* ≤ 0.05). ^x, y^ Means without a common superscript are different by pairwise comparison (*p* ≤ 0.10).

**Table 3:** The health responses of Holstein steers supplemented with either a proprietary tannin-blend additive (SBPX; 14,500 mg/hd Silvafeed ByProX) or monensin (MON; 320 mg/hd Rumensin™ 90).

	Treatment			
	MON	SBPX	SEM ^1^	*p*-Value ^2^
Pens, *n*	10	10		
Head per pen	72	73		
Total Morbidity, %	25.49	26.03	2.987	0.82
First Treatment Protocol, %	22.77	23.79	2.508	0.65
First Pull, %	15.49	17.62	1.760	0.27
Second Pull, %	4.94	4.96	1.043	0.98
Third Pull, %	1.97 ^x^	0.96 ^y^	0.643	0.10
Second Treatment Protocol, %	1.79	1.63	0.581	0.80
Third Treatment Protocol, %	0.79	0.55	0.393	0.54
Treatment cost, USD/hd	10.08	8.50	1.660	0.35
Bullers, %	5.91	5.75	1.141	0.90
Railers, %	0.70	0.69	0.313	0.97
Total Mortality, %	1.54	2.11	0.612	0.41

^1^ The largest standard error of the mean. ^2^ Tested as the ANOVA significance of the main effect. ^x, y^ Means without a common superscript are different by pairwise comparison (*p* ≤ 0.10).

**Table 4:** Carcass characteristics of Holstein steers supplemented with either a proprietary tannin-blend additive (SBPX; 14,500 mg/hd Silvafeed ByProX) or monensin (MON; 320 mg/hd Rumensin™ 90) after 358 ± 2 days on feed.

	Treatment			
	MON	SBPX	SEM ^1^	*p*-Value ^2^
Hot carcass weight, kg	362.3	363.9	2.08	0.53
12th rib fat, cm.	0.95	0.95	0.025	0.90
Ribeye area, sq. cm.	74.94	74.39	0.643	0.44
Quality grade distribution ^3^				0.22
Prime, %	13.25	10.94	1.346	0.20
Choice, %	84.23	86.32	1.448	0.29
Select, %	1.42	2.02	0.701	0.37
Standard, %	0.79	0.30	0.351	0.25
Yield grade distribution ^3^				0.79
USDA YG 1, %	2.20	2.42	0.599	0.79
USDA YG 2, %	8.07	6.44	1.797	0.23
USDA YG 3, %	88.56	90.24	1.930	0.31
USDA YG 4, %	0.25	0.24	0.252	0.97
USDA YG 5, %	0.32	0.00	0.222	0.99
Total liver abscess, %	10.08	12.27	1.277	0.21

^1^ The largest standard error of the mean. ^2^ Tested as the ANOVA significance of the main effect. ^3^ Tested using ordinal logistic regression.

**Table 5:** Polyphenol status (μg/100 g muscle) of Holstein steers supplemented with either a proprietary tannin-blend additive (SBPX; 14,500 mg/hd Silvafeed ByProX) or monensin (MON; 320 mg/hd Rumensin™ 90) after 358 ± 2 days on feed.

		Treatment			
	Polyphenol ^1^	MON	SBPX	SEM ^2^	*p*-Value ^3^
Alkaloids	Hordenine	25.48	75.75	32.42	0.05
Flavonoids	Epicatechin gallate	0.04	0.001	0.03	0.25
	Epigallocatechin gallate	0.04	<LOQ	0.01	<0.01
	Apigenin	1.53	0.71	1.30	0.04
	Chrysoeriol	0.34	0.02	0.25	0.02
	Diosmetin	0.38	0.05	0.26	0.06
	Apiin	1.78	0.63	0.88	0.52
	Isovitexin	0.42	0.07	0.28	0.16
	Linarin	1.25	0.20	0.84	0.10
	Orientin	0.32	<LOQ	0.23	<0.01
	Schaftoside	4.15	0.84	2.90	0.16
	Sophoricoside	0.72	0.13	0.06	<0.01
	Hyperoside	2.2	1.99	0.07	0.10
	Narcissoside	0.82	0.12	0.56	0.43
	Rutin	1.48	0.06	0.95	0.09
	Isorhamnetin	2.81	21.74	13.22	0.17
	Kaempferide	0.21	<LOQ	0.17	<0.01
Phenolic acids	Gallic acid	0.09	0.01	0.04	0.57
	2,6-Dihydroxybenzoic acid	0.06	<LOQ	0.03	<0.01
	3,4-Dihydroxybenzoic acid	0.04	0.05	0.02	0.04
	4-Hydroxybenzoic acid	2.91	2.33	0.47	0.65
	Syringic acid	0.04	0.03	0.02	0.52
	Vanillic acid	0.28	0.14	0.11	0.42
	Caffeic acid	0.49	0.08	0.00	<0.01
	Chlorogenic acid	0.17	0.01	0.09	<0.01
	p-Coumaric acid	0.62	0.14	0.19	0.01
	trans Ferulic acid	0.15	0.003	0.09	0.11
	Salicylic acid	0.29	0.14	0.05	0.03
	4-Ethylphenol	10.26	5.37	4.08	0.02
Gut-derived phenolic metabolites	Catechol sulfate	0.62	0.83	0.14	0.39
	p-Cresol sulfate	42.02	51.37	9.04	0.63
	Hippuric acid	22.50	29.73	6.66	0.52
	Dimethyl Sulfone	2.40	3.72	0.77	0.33
Others	Ectoine	0.09	1.94	1.60	0.06
	Ergothioneine	5.87	3.08	1.45	0.28
	Hercynine	15.35	29.63	12.17	0.65
	Hypaphorine	4.78	1.62	1.23	0.04
	Isocitric lactone	6.88	6.10	1.44	0.80
	Imperatorin	0.80	0.11	0.65	0.06
	Morin	0.07	1.83	1.49	0.63
	Trigonelline	2.31	3.56	1.31	0.44
	Tyrosol	0.15	0.08	0.03	0.04

^1^ Information about individual metabolites can be sourced from the Human Metabolome Database (HMDB). ^2^ The largest standard error of the mean. ^3^ Tested as the ANOVA significance of the main effect. <LOQ: below the lowest level of quantification.

## Data Availability

The raw data supporting the conclusions of this article will be made available by the authors on request.
